# Straw phytolith for less hazardous open burning of paddy straw

**DOI:** 10.1038/s41598-019-56735-x

**Published:** 2019-12-27

**Authors:** Anh T. Q. Nguyen, Minh N. Nguyen

**Affiliations:** 1 Faculty of Environmental Sciences, VNU University of Science, Vietnam National University, Hanoi (VNU), 334 Nguyen Trai, Thanh Xuan, Hanoi Viet Nam; 2 Faculty of Biology, Thai Nguyen University of Education, Thai Nguyen University (TNU), 20 Luong Ngoc Quyen, Thai Nguyen, Viet Nam

**Keywords:** Environmental sciences, Environmental chemistry, Environmental impact

## Abstract

Rice production helps feed at least half of the world’s population but generates approximately one billion tonnes of straw residue per annum. On-site open burning of rice straw after harvesting is common in recent times because there has been less demand for rice straw to use as fuel and fodder. Due to health and climate change concerns, open burning, which results in biomass losses, smog and emissions of green house gases, e.g., CO_2_, has been widely criticized and banned in many countries. Little is known about the positive benefits of straw burning, such as field care (eradication of biotic diseases) or nutrient cycling. Herein, we propose a new viewpoint in which the burning of rice straw followed by cycling of the burned materials, including silica material (so-called phytolith), into soil is demonstrated as a CO_2_-sequestration strategy via buffering the soil CO_2_ flux and coupling CO_2_ with the silicon cycle.

## Introduction

Rice straw burning has been widely judged as an environmental concern contributing to global CO_2_ and black carbon emissions^1^. Many regions or countries, e.g., the US, EU, China, India, Australia and Southeast Asia, have banned straw burning, although these bans were likely based on a singular view that is still under debate^2–4^. Even so, immediate cessation of on-site burning worldwide is implausible because there is lack of consensus in many rice-based countries, where the governments cannot involve farmers in alternative practices that are more effective^3^. This outcome suggests that such bans need stronger evidence or incentives to cause farmers to change their method of handling straw. Any incentives towards reducing or stopping on-site burns will likely reshape the method of managing straw, affecting hundreds of millions of farmers worldwide. Currently, the best method is unknown because straw burning is not simply an environmental treatment. We also have to address other relevant issues, such as agronomic re-practicing, soil degradation, disease outbreaks and the increasing accumulation of arsenic in rice. These issues indicate that a multi-dimensional approach or integrated solution is crucial for solving straw burning issues and that a balance between “gains and losses” needs to be strictly taken into account.

Mitigation of CO_2_ emissions is a common reason for bans; however, this adjudication is placed in a relatively narrow context – “straw biomass loss from burning”, and the effect of the burning process on the whole CO_2_ cycle of the paddy rice system is neglected. Recent studies have revealed that burning removes straw organic carbon (OC) but also releases burned matter (alkaline elements and fast-reacting silica) that have been reported to increase CO_2_ sequestration and reduce CO_2_ emissions from soil^5,6^. In addition, straw biomass is routinely believed to be fully decomposed in open burning, while the effect of other inter-embedded matter, e.g., phytolith, is ignored. Many recent works have demonstrated the phytolith structure is a carrier that sequesters straw OC^7–9^, and some studies have reported the possible ability of phytoliths to act as a shield to slow down the thermal decomposition of OC^10,11^. Obviously, this result encourages further work on how and to what extent phytoliths can preserve straw OC under open burning. Our research with a central focus on CO_2_ and phytolith interactions was to demonstrate the contrasting scenarios of burning rice straw and CO_2_ emission/sequestration and to show the need to review both the negative and positive aspects of the straw open burning before enforcing hard bans.

## Results

### Straw phytolith captures organic carbon

**Figure 1:**
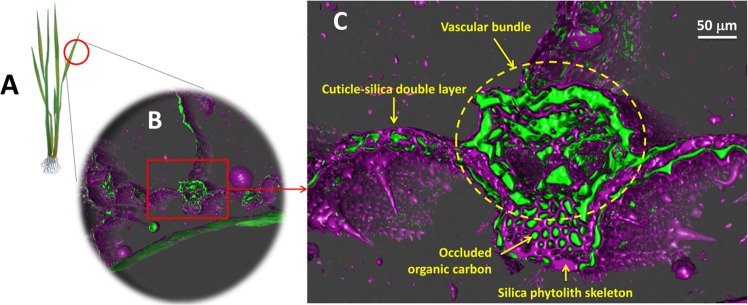
Occlusion of organic carbon in rice straw phytoliths: **(A)** rice plant, **(B)** X-ray tomographic image showing the rice leaf, and **(C)** a part of the leaf containing the silica phytolith skeleton (coloured in violet) and subcompartmentated organic matter (coloured in green).

Phytolith is a silica structure that is commonly observed in silicon (Si)-rich plant species, e.g., grass, bamboo, ferns, wheat and especially rice^12^. Tomographic observations of rice straw have illuminated a phytolith structure and its subcompartmentation of OC (Fig. 1A–C). Within the bundle sheath and the leaf surface, tightly packed bundle-sheath cells and more loosely arranged mesophyll cells are held by silicified structures in inter- and intra-cellular spaces. Condensation of the silica phase in the leaf part is most likely due to the abundance of Si input supplied from transport sap and a high evaporation rate^13,14^. Forming inside organic tissues, phytolith is fed by the polymerization of Si, leading to an expansion of its legacy and eventually occlusion of OC in its structure. As a result, OC is involved in the fate of phytoliths. When plant residues are cycled into soil, phytolith-occluded OC (phytOC) can also be cycled and accumulated in soil. This phytOC is preserved by the phytolith structure and is secure from biodegradation.

To date, the occlusion of OC within the phytolith structure has been examined in many studies^7,8,15^. A recent high-end technique, i.e., X-ray microtomography, allows visualization inside the tissue organs of straw and provides a better understanding of how the silica phytolith skeleton can protect the OC occluded inside its structure (Fig. 1C). The association of phytolith silica and occluded OC can be physically and chemically strengthened. Condensation of phytolith silica via precipitation of Si from transport sap is a process that can consolidate the physical association between the phytolith and cuticle at the outer tangential epidermis cell wall^16^. Chemical bonds such as Si−C and Si−OR form on the tissue cell walls as a result of the priming effects of the chemical association followed by dehydration of silica and organic carbon^17^. This physico-chemical reaction chain strengthens the occlusion of OC within the phytolith structure.

### Thermal resistance of phytolith-associated organic carbon

**Figure 2:**
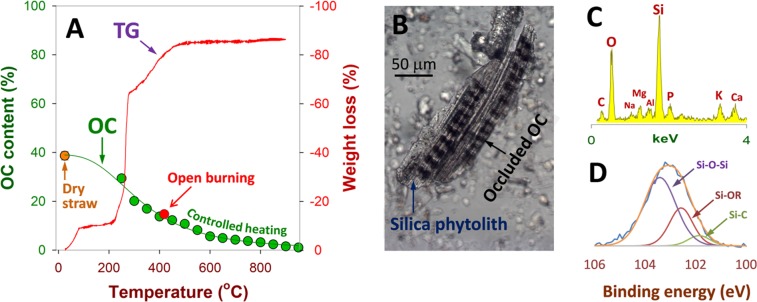
Thermal resistance of phytolith occluded OC: (**A)** plots of weight loss and OC content upon increases in heating temperature, by which some thermal recalcitrant OC is indicated; (**B)** a microscopic image showing an open-burning derived phytolith fragment and its occluded OC; **(C)** EDX spectra representing the surface chemical composition of a phytolith fragment; and **(D)** the typical Si 2p XPS survey spectra of a phytolith fragment (R refers to any chain of carbon atoms (alkyl group) or a single hydrogen atom).

In rice paddy systems, open burning rather than incorporation is the preferential practice, and this process can alter both phytoliths and their occluded OC^18,19^. Phytoliths will be reshaped because silica is subject to condensation to form more stable phases, e.g., cristobalite or tridymite^20^, whereas OC will be transformed eventually into carbon gases, e.g., CO_2_, CO, C_1_−C_2_ hydrocarbons and other volatile substances^21^. Figure 2A clearly shows the weight loss along with the decrease in the OC content. While open burning at approximately 420 °C resulted in a loss of half the OC, temperatures at >400 °C (in controlled pyrolysis experiments) led to more extensive loss of OC. The outer OC (outside the phytolith structure), which is directly subjected to fire or high temperatures, will be removed first. The inner OC (here phytOC) might be decomposed at lower rates, and the extent to which phytOC is lost by thermal decomposition is likely related to the strength of its association with the phytolith structure. If it is protected by the phytolith structure, where limited oxygen can penetrate, phytOC is believed to be more recalcitrant to thermal decomposition, and consequently black carbon can be formed to a given extent. The dark parts inside straw ash from open burning (easily observable by naked eyes) might be related to the black carbon derived from the internal anaerobic conditions of the ash phytolith structure. In ash phytoliths, black carbon is commonly observed in association with the silica phytolith structure (Fig. 2B,C). The black carbon or internal OC part is protected by the associating phytolith and stabilised by forming Si−C and Si−OR bonds with phytolith silica (as deduced from XPS analyses in Fig. 2D).

### Straw-derived phytoliths are involved in buffering soil CO_2_ fluxes

**Figure 3:**
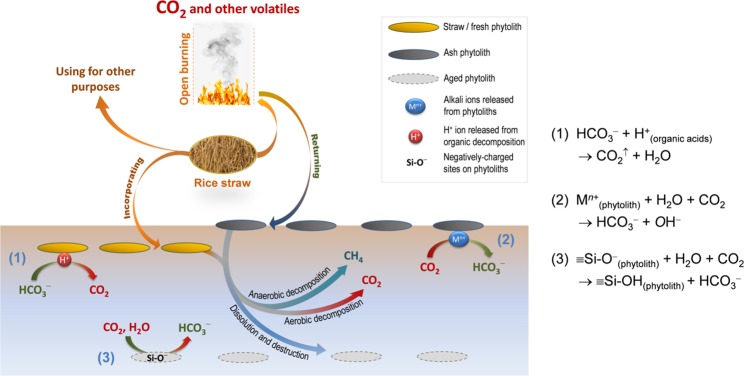
Schematic description of CO_2_ flux in a rice paddy system. Three possible processes of CO_2_ conversion that are driven by fresh, ash or aged phytoliths.

Phytolith makes up from 1.6 to 14.4% of rice (stem, sheath, and leaf)^9,22^, thereby hundreds of million tonnes of phytolith are recycled annually if all world rice residues are returned to the soil instead of exported or used for other purposes. Phytoliths, including fresh phytolith (from straw residue incorporated into soil), ash phytolith (obtained from straw burning) and aged phytoliths (both fresh and ash phytoliths buried in soil), might co-exist in rice field. Because CO_2_ fluxes (as high as 8.62 µmol m^−2^ s^−1^ ^23^) originate from the aerobic decomposition of soil OC (e.g., in root zone) and can diffuse extensively over a rice field, it is likely that CO_2_ fluxes penetrate water and contact phytoliths, by which they are then transformed in different ways, as schematically described in Fig. 3.

The solubility of CO_2_ is highly pH dependent, and the soluble forms of CO_2_ include H_2_CO_3_, HCO_3_^−^ and CO_3_^2^^−^. Therefore, the straw cycle and its derived phytoliths in the soil can change the soil pH and drive the CO_2_ buffer capacity. Incorporation of straw into soil has been encouraged^24^, but it creates a more acidic environment because straw serves as organic matter for decomposition to form organic acids^25^. Hence, this practice, as a result, decreases the CO_2_ buffer capacity and favours CO_2_ emission (reaction #1). In contrast, open burning removes organic matter (transformed to gases) and produces highly alkaline materials with typical pH values ranging from 10 to 11^13^. Burning and returning burned products (ashes) can generally increase the soil pH, leading to higher solubility of CO_2_. Ashes contain not only immediately available alkali ions (Supplementary Table S1) but also phytolith-occluded alkali ions^13^. Considering these outer and inner alkali ion sources, the effect of burned materials in buffering the soil CO_2_ flux can be divided into two stages involving the direct release of outer alkali ions and the “slow release” of phytolith-occluded alkali ions (reaction #2). In an experiment to simulate the release of potassium (K) accompanying the dissolution of phytoliths, Nguyen, *et al*.^20^ demonstrated a mechanism in which the K located in the phytolith structure is slowly released when the phytoliths are destroyed. This result implies that ash phytoliths can increase the soil pH and buffer CO_2_ flux via releasing and providing their internal alkali elements. Dissolution of silicate minerals has been known as a natural process that captures CO_2_^6^. This CO_2_ capture can be magnified for fast-reacting silicate rocks (e.g., basalt)^5,26^. Likewise, phytolith silica, which is a highly soluble material^13^, is also expected to react in the same way to mitigate CO_2_ emission. Because the dissolution rate of phytoliths is controlled by various physico-chemical properties of soil solutions, e.g., pH and ionic strength^13^, the fate of phytoliths and their role in mitigating CO_2_ emissions are, therefore, expected to vary from one soil/region to another.

**Figure 4:**
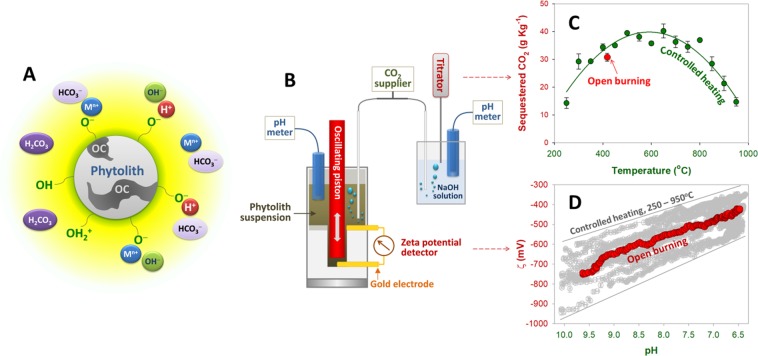
CO_2_ conversion driven by the negative surface of phytoliths and alkali soluble ions released from phytoliths: **(A)** A schematic description regarding the co-presence of phytolith, its occluded OC and other soluble substances in solution; **(B)** a parallel reactor system designed to examine the effect of increasing CO_2_ concentration on: (**C**) CO_2_ sequestration and **(D)** change in the surface charge (zeta potential, ζ) of the suspensions prepared from phytoliths obtained from open burning and controlled heating in a temperature range from 250 to 950 °C.

The second CO_2_ sequestration mechanism (reaction #3) relates to electro-chemical interactions between CO_2_ and phytolith surfaces (Fig. 4A). Our parallel system to measure CO_2_ adsorption and the electro-kinetics of phytolith suspensions (Fig. 4B) revealed the ability of phytoliths to capture CO_2_ (Fig. 4C) (Supplementary Table S2) and the electro-chemical process that governs CO_2_ sequestration (Fig. 4D). In a suspension, the phytolith surface develops a net negative charge primarily due to deprotonation of the surface Si−OH groups, and this process steadily increases in the pH range from 2 to 10 or even higher^13,27^. Reversibly, protonation of the negative phytolith surface might occur, and this process is likely a highly rapid, exothermic reaction with no significant activation energy that occurs via the concerted motion of the protons along a chain of water molecules^27^. Because H_2_CO_3_ acid has a pK_a1_ of 6.4, we can expect phytoliths with their alkaline nature to facilitate the deprotonation of H_2_CO_3_ and attract free H^+^ ions for transfer to their surface, by which phytoliths facilitate CO_2_ conversion into HCO_3_^−^ or even CO_3_^2−^ and prevent reversion of CO_3_^2−^ or HCO_3_^−^ back to CO_2_. In our short-run experiments, the protonation due to the increasing presence of CO_2_ in suspensions resulted in gradual decreases in the pH and increases in zeta potential (ζ) (Fig. 4D) (Supplementary Table S3). Differences in the variation ranges of pH and ζ between the open burning and controlled heating samples were likely because of their charge densities^28^. The CO_2_ amounts that reacted with the phytolith suspensions were calculated to be ~30g CO_2_ kg^−1^ for the open burning sample and from ~15 to 40g CO_2_ kg^−1^ for the controlled heating samples.

## Discussion

The findings on the positive aspects of on-site burning with the central role of phytoliths suggest a need for multi-dimensional views to mediate the debates and encourage new strategies. By bringing CO_2_ emission from on-site straw burning and CO_2_ sequestration in soils driven by straw-derived phytoliths together, a more detailed picture of the gains and losses from open burning was revealed. Straw open burning results in direct CO_2_ emission; however, the burned products remaining in a field can be involved in processes that enable CO_2_ sequestration via conversion of CO_2_ into HCO_3_^−^ or CO_3_^2−^ in the presence of phytoliths. This conversion might be related to various processes, such as the dissolution of straw ash phytoliths to release alkali elements, the reaction of released alkali elements and dissolved CO_2_ to form HCO_3_^−^, and protonation of the negatively charged surface of phytoliths that can convert H_2_CO_3_ into HCO_3_^−^. All these processes occur concurrently and interactively; however, our study still cannot determine the order, rate and preference of each individual process.

In addition, it was evidenced that the phytolith skeleton can also protect its occluded OC by favouring an “internal anaerobic” condition, which prohibits oxidation of OC and reduces CO_2_ emission from open burning. This result suggests that developments in burning techniques considering electro-chemical changes can both preserve straw OC and produce materials to enhance soil CO_2_ sequestration. Overall, as straw management might involve billion tonnes of CO_2_ annually released or sequestered worldwide, it is urgent to re-evaluate straw burning on the field scale at which elemental cycles can be entirely closed and other benefits from burning rice straw can also be assessed. More extensive and intensive discussion is necessary. Biomass loss and air pollution are the major reasons for exporting straw, and not returning straw’s occluded nutrients to a field need to be re-considered. Straw ash phytoliths should be given a chance to demonstrate their potential to mitigate soil CO_2_ emissions.

Our short-run experiments indicated possible reactions that sequester CO_2_ in phytolith suspensions; however, these experiments cannot simulate or generalize the effect of phytoliths on CO_2_ emissions on field, regional or larger scales, where many challenges will be raised. The heterogeneous dissolution of phytoliths due to their complicated chemical composition is one of the most difficult obstacles, and many attempts are still being made to elucidate the ageing of phytoliths and the subsequent changes in their surface properties. Under field conditions, soil biological processes, e.g., decomposition, mineralization, and respiration, which may vary from one region to another, will likely alter phytoliths and their surface reactions to sequester CO_2_ at different levels. Therefore, the inclusion of such individual experiments from various ecoregions or biomes will allow the creation of larger and more realistic databases for the calculation of CO_2_ sequestration. Last, many current cultivation initiatives regarding fertilization, irrigation, puddling, and weeding have been strategically moved towards decreasing CH_4_ emissions or limiting arsenic accumulation in rice, and from now, in addition to CH_4_ and arsenic issues, it is also worth incentivizing methods that can activate phytoliths to sequester more CO_2_. If all these challenges are solved, adjustment of the contributions or effects of straw phytoliths to the global CO_2_ cycle can be made.

## Materials and Methods

### Sample preparation

Straw samples were collected at harvest time in a central part of the Red River Delta, Vietnam. Samples were air dried, oven dried at 70 °C, and then finely chopped into ~1 mm segments prior to analysis. Open burning of each 500 g of straw was performed in open-air conditions, and it took approximately 20 min for the straw samples to be burned completely. The burning temperatures, which were measured by directly injecting a thermo probe (330-1 LL, Testo) into the straw piles, were averaged to be ca. 420 °C. Because burning is exothermic depending upon the ambient air condition (e.g., wind velocity, humidity) and the manner of burning (scatter or pile up), we also treated straw samples in controlled pyrolysis conditions to evaluate the chemical composition and surface properties of the obtained straw phytoliths over a wider temperature range. The pyrolysis process was set up at a heating rate of 10 °C min^−1^ and maintained at the target temperatures (from 250 to 950 °C with intervals of 50 °C) for 1 h. The samples obtained from open-burning and controlled pyrolysis conditions were ball milled and passed through a 0.25 mm sieve.

### Sample characterization

The chemical composition of the straw and ashes obtained from open-burning and controlled heating experiments was examined by the PIXE (particle induced X-ray emission) method, using the proton beam of the Tandem accelerator (5SDH-2 Pelletron accelerator system, manufactured by National Electrostatics Corporation, USA) (Supplementary Table S4). The OC content in the phytoliths was determined using the classical wet digestion method with concentrated H_2_SO_4_ and 133 mol L^−1^ K_2_Cr_2_O_7_ at 170–180 °C^29^. Thermogravimetric analysis (TGA) was conducted for straw samples using a Macro-TGA device designed by CIRAD. Specific surface area (SSA) was measured using a Gemini VII 2390p surface area analyser. Soluble ions dissolved from open-burning or controlled heating ashes were analysed by using inductively coupled plasma mass spectrometry (ICP-MS 7900, Agilent Technologies). The accuracy was assessed using the European Commission’s standard reference materials BCR-277R. Recovery rates of the elements ranged from 90% to 110% (Supplementary Table S1).

The straw sample was scanned by ZEISS Xradia 520 Versa 3D X-ray microscope at the University of Kassel. The 3D X-ray microscope enables visualization of the silica phytolith structure and organic matter phase in rice leaves. The 3D phytolith and organic matter structures were assembled from 975 individual 2D image slices with 995 × 972 isotropic pixels (the pixel size was 1.14 µm) using the open-source software YaDiV^30^. The 256 grey values depict element densities: air has grey values < 50), the OC phase coloured in green has grey values from 50 to 200, and the high-density phase, i.e., phytolith, coloured in violet has grey values of approximately >200. X-ray photoelectron spectroscopy (XPS) is a particular surface-sensitive analysis technique, and the XPS-based data can be expected to visualize the integration between organic matter and inorganic phases (e.g., phytolith). The open-burning ash sample was analysed by XPS using an AXIS-NOVA spectrometer (SHIMADZU/KRATOS, Japan) with monochromatic AlKa radiation (15 kV 10 mA).

### Electrokinetics of phytoliths and CO_2_ sequestration

The parallel and connected systems assembled to monitor the electrokinetics of phytoliths and CO_2_ sequestration are illustrated in Fig. 4B. CO_2_ gas was supplied at the rate of 0.1 L min^−1^ into two parallel reactors for (1) monitoring ζ changes and (2) measuring reacted CO_2_ (adsorbed) at the same time. In the first block, ash phytolith suspensions (50 mg in 20 mL deionized water) were transferred into a reaction Teflon cup in the PCD-based system with a particle charge detector (PCD 05, Muetek), and the supply of CO_2_ was halted when the pH stopped decreasing. Changes in pH and ζ were recorded every 10 s, and the experiments were terminated when the pH stopped decreasing. In the second block, CO_2_ was consumed (adsorbed) in 20 mL of a 0.005 N NaOH solution, which represents CO_2_ reacting with phytolith suspensions (in the first block), and titrated with 0.005 N H_2_SO_4_ solution. All measurements were conducted in triplicate. NaOH and H_2_SO_4_ were purchased from Merck, while CO_2_ (99.99%) was provided by Ha Bac Nitrogenous Fertilizer and Chemical Ltd. Company.

## Supplementary information

Supplementary file.
